# Should thorough Debridement be used in Fibular Allograft with impaction bone grafting to treat Femoral Head Necrosis: a biomechanical evaluation

**DOI:** 10.1186/s12891-015-0593-3

**Published:** 2015-06-10

**Authors:** Guangquan Zhou, Ying Zhang, Linghong Zeng, Wei He, Zhihui Pang, Xiumin Chen, Yujing Xu, Liao Shaoyi Stephen, LeiLei Chen

**Affiliations:** The First Affiliated Hospital, Guangzhou University of Chinese Medicine, Guangzhou, China; Laboratory of National Key Discipline Orthopaedics and Traumatology of Chinese Medicine, Guangzhou University of Chinese Medicine, Guangzhou, China; Department of Rheumatology, Guangdong Provincial Hospital of Chinese Medicine, China and Postdoctoral Mobile Research Station, Guangzhou university of Chinese Medicine, Guangzhou, China; Luoyang Orthopedic-Traumatological Hospital, Henan, China; Department of Rehabilitation Medicine, The 3rd People’s Hospital, Huizhou, China 516002; Department of Information Systems, City University of Hong Kong, Hong Kong, China

**Keywords:** Computational biomechanics, Thorough debridement, Stress transfer path, Load share ratios, Stress shielding

## Abstract

**Background:**

Fibular allograft with impaction bone grafting (FAIBG) is an effective hip-preservation method for avoiding total hip arthroplasty in the early stage of femoral head necrosis. However, whether thorough debridement should be used with FAIBG is controversial. This study compared the mechanical performance between FAIBG with and without thorough debridement, which provides a biomechanical basis for selecting the proper treatment in clinical settings.

**Methods:**

Eighteen computational models were constructed and used to simulate two subtypes of femoral head collapse with seven debridement radii. The initial model was validated using the bony density distribution from X-ray images and a photograph of the cadaver bone cross-section. The stress of the anterolateral column and the debridement efficiency were computed and analyzed.

**Results:**

(1) The peak stress of the anterolateral column in all conditions could return to the physiological level, and in two cases, the decrement/increment of stress was almost less than 0.1 % when the debridement radius increased. (2) The load share ratio (LSR) of the cortical and cancellous bone was markedly decreased in the untreated condition and increases with an increase in the debridement radius. (3) A debridement radius greater than 1/2r yields a LSR value larger than that obtained in the normal condition.

**Conclusions:**

The simulation results provide specific biomechanical evidence to support the finding that FAIBG with a debridement region of 3/8 -1/2 appears to be a better choice for resisting femoral head collapse (FHC). Furthermore, FAIBG without thorough debridement, which requires relatively simple surgical devices and reduces artificial damage, appears to be a better method for resisting FHC than FAIBG with thorough debridement.

## Background

The incidence of femoral head necrosis (FHN) is rapidly increasing worldwide because of the widespread use of steroids [1, 2] and alcohol [3–6]. FHN is associated with high morbidity and disability. Patients with FHN are often at high risk of femoral head collapse (FHC), arthritis or disarticulation, which finally results in hip replacement (HR). Statistical data show that the medium- and long-term effects of hip-implant are obviously unsatisfactory; thus, young patients with HR will require several surgical treatments [7]. Hence, various head-preserving procedures have been developed to protect the femoral head of patients and avoid HR, particularly in the early stage of FHN.

Fibular allograft with impaction bone grafting (FAIBG) is an effective head-preservation method for avoiding HR in the early stage of FHN. The advantage of this hip-preservation method is that it provides both repaired materials and biomechanical structural support during the healing of the necrosis region [8–11]. However, the disadvantage in using the FAIBG procedure lies in the fact that wide debridement may increase the incidence of cartilage injury and the strength of impaction bone grafting is difficult master. Hence, whether thorough debridement should be used with FAIBG is controversial. “With thorough debridement” indicates that the necrotic bone should be completely cleaned, whereas “without thorough debridement” indicates that the necrotic bone should undergo partial debridement. In most cases, the choice is based on the experience and preference of the surgeon without scientific evidence. Simultaneously, relatively few studies have compared the risk of postoperative FHC with and without thorough debridement.

The clinical practice concept requires theoretical proof. This study presents two subject-specific FHN cases without FHC to compare the mechanical performance between FAIBG with and without thorough debridement, which provides a biomechanical basis for selecting the proper treatment in clinical settings.

## Methods

### JIC Classification

In 2001, the Japanese Investigation Committee (JIC) [12] revised the diagnostic criteria used to clarify the definition of osteonecrosis of the femoral head (ONFH). According to the JIC classification criteria, FHN is classified into subtypes A, B, C1 and C2 based on the location of the lesion in the weight-bearing area. Type A lesions occupy the medial one-third or less of the weight-bearing portion, type B lesions occupy the medial two-thirds or less of the weight-bearing portion, type C1 lesions occupy more than the medial two thirds of the weight-bearing portion but do not extend laterally to the acetabular edge, and type C2 lesions occupy more than the medial two-thirds of the weight-bearing portion and extend laterally to the acetabular edge.

**Fig. 1:**
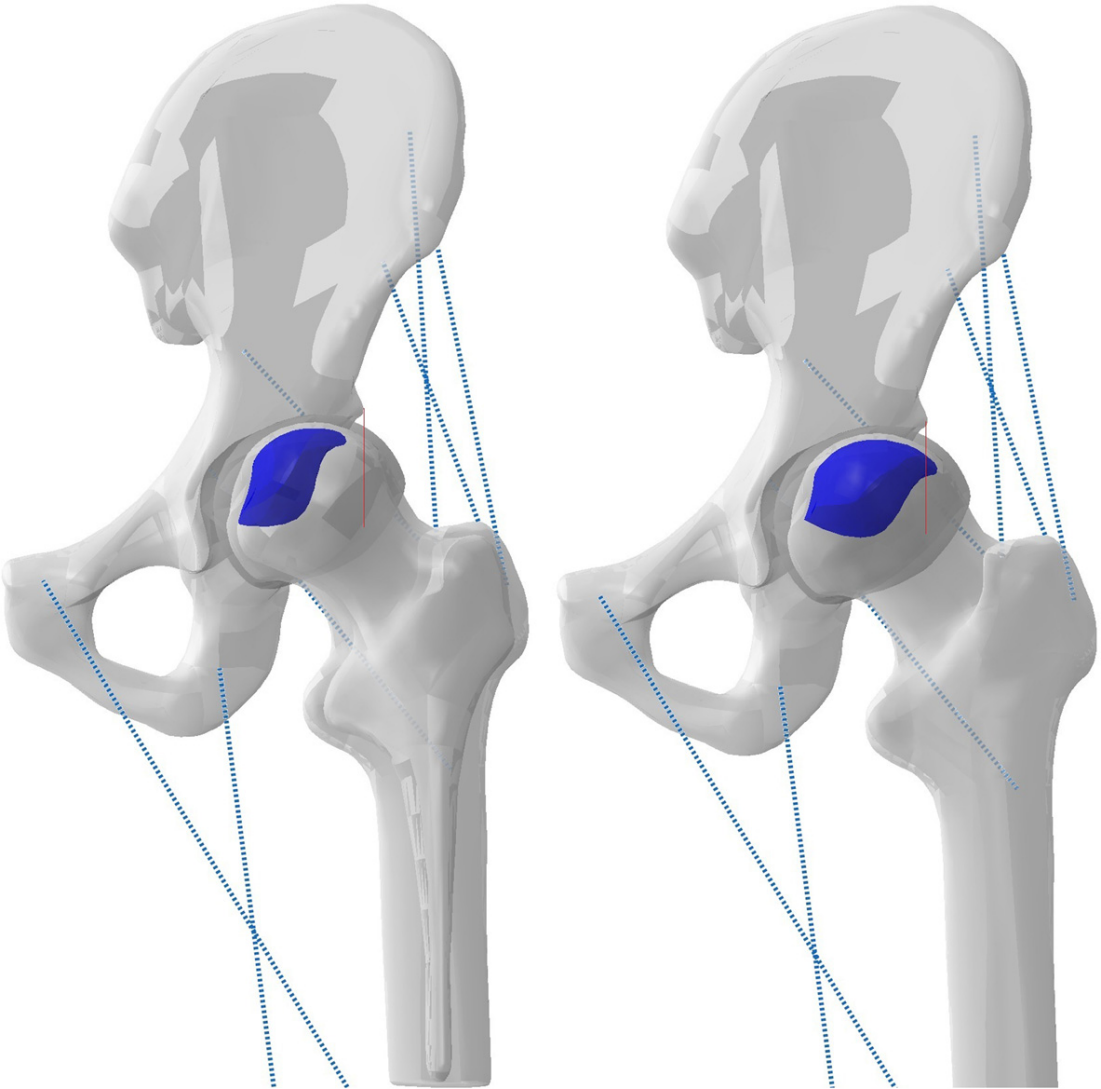
3D subtype models of FHN

Recent studies show that patients who conform to the JIC C criteria are suitable for FAIBG. However, these conclusions are mainly based on clinical observation experience and must be proven both theoretically and in practice. Whether thorough debridement should be used with FAIBG remains controversial. We postulated that the FAIBG procedure with different debridement regions results in different biomechanical performances, which could affect the choice of treatment procedure for FHN. Hence, we reconstructed two subject-specific models (JIC C1 and C2, Fig. 1) to provide a biomechanical basis for exploring the performance of FAIBH with different debridement regions for the treatment of FHN [Ethical approval was granted by the local ethics committee (Constitution of the medical ethics committee: The First Affiliated Hospital, Guangzhou University of Chinese Medicine). Written informed consent for participation in the study was obtained from participants.].

### Generation of Intact Finite Element Models

A JIC C1 FHN-diagnosed patient (P1, last name Fu) with a weight of 70 kg and a JIC C2 FHN-diagnosed patient (P2, last name Wan) with a weight of 60 kg were selected for the biomechanical evaluation of the proximal femur (informed consent was obtained from all patients). Computed tomography datasets (0.5 mm thickness; Toshiba Aquilion 64, Japan) for each case were used to reconstruct solid models with grey-level processing with the MIMICS 15.1 software based on the “Thresholding”, “Edit Masks”, and “Calculate 3D” functions. The solid models in the STL format were inputted into the Rapidform pre-processor, and surface-fitting was then performed. Based on the “Mesh” and “Autosurfacing” functions, we found the fit hip to generate the NURBS models. The interface between the ilium and femoral head was used to identify the cartilage geometry. All NURBS models in the igs format were inputted into ABAQUS V6.13 (SIMULIA co., France) to generate nonlinear elastic finite element models. Based on the initial hip geometry, we simulated physiological and pathological models using different materials.

**Fig. 2:**
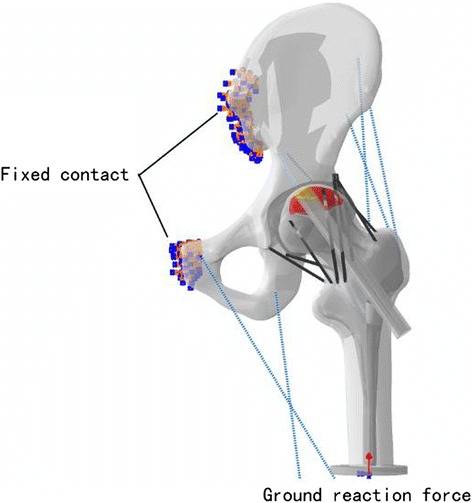
Load and constraint conditions

All of the models were then inputted into ABAQUS V6.13 to generate isotropic 10-node tetrahedral elements with a mesh size of 4 mm. The initial models consisted of various elements (146879 in P1; 156471 in P2) and nodes (213970 in P1; 230541 in P2). In these models, the single-legged stance was considered a representative body position, and a ground reaction force equivalent to the body weight was performed on a rigid plate, which was tied to the distal part of the femur in Fig. 2. Constraints were applied to the pubic symphysis and sacroiliac joint. All six degrees of freedom were constrained to zero. Seven muscles were modeled as axial connectors, and the muscle forces were set according to the literature [13]: adductor longus = 560 N; adductor magnus = 600 N; gluteal maximus = 550 N; gluteal medius = 700 N; gluteal minimus = 300 N; piriformis = 500 N; tensor fascia latae = 300 N. The models consist of cortical, trabeculae, cartilage and lesion bone. The material properties used in the biomechanical experiment were obtained from the literature [14–16]: Ecortical = 15,100 MPa, Etrabeculae = 445 MPa, Ecartilage = 10.5 MPa, Elesion = 124.6 MPa, νcortical = 0.3, νtrabeculae = 0.22, νcartilage = 0.45 and νlesion = 0.152.

**Fig. 3:**
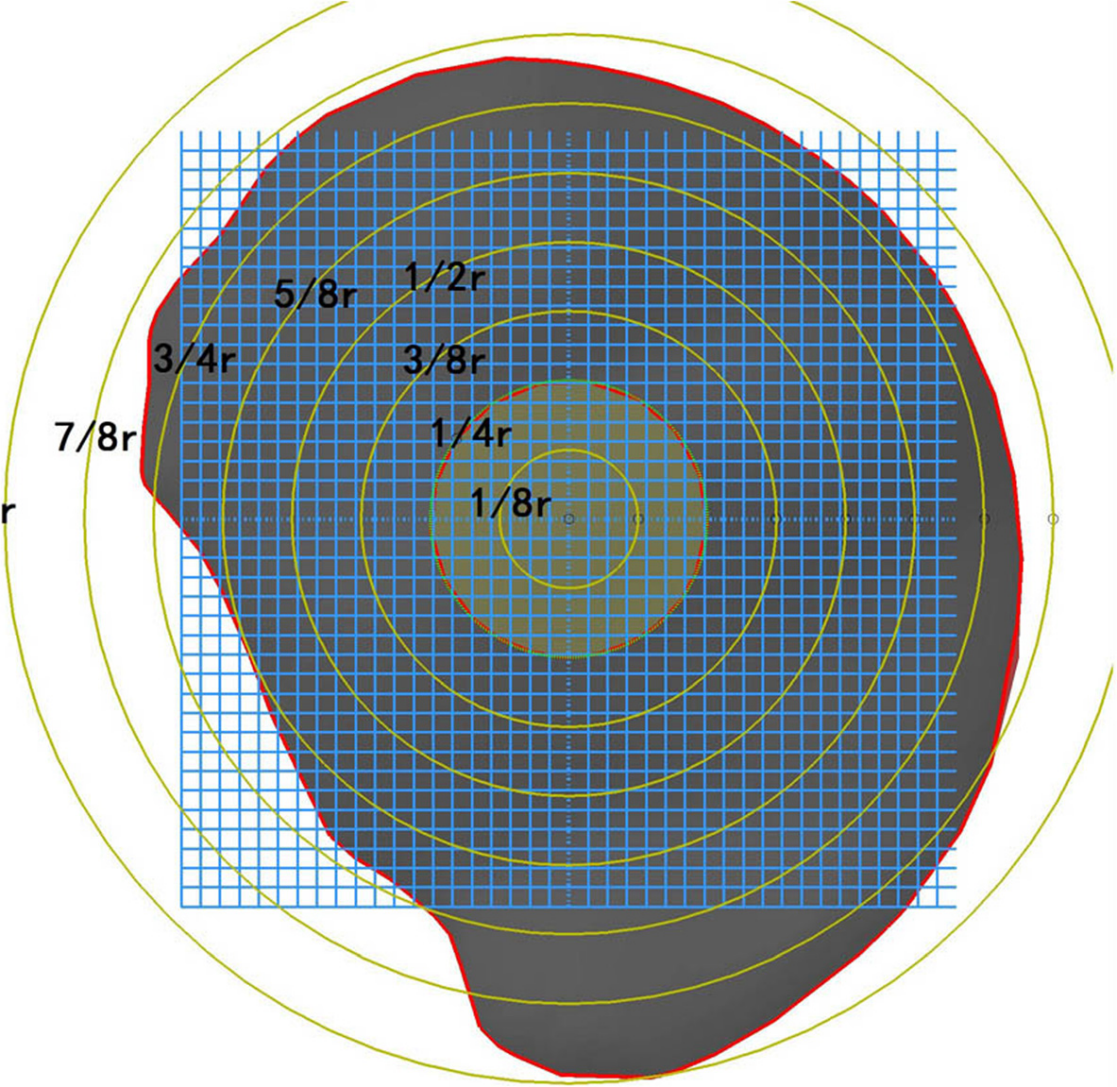
Debridement size of necrotic lesion

The parametric analysis was designed to explore the effects of the extent of debridement of necrotic bone in cases that require surgery. The maximum debridement radius was defined as r, and the debridement extent variants are schematically shown in Fig. 3. We assumed that the anterolateral cortical stress corresponding to the debridement extent of the necrotic lesion had an increased radius R (R = 1/4 r, 3/8 r, 1/2 r, 5/8 r, 3/4 r, 7/8 r and r), where R = 1/4r refers to the least debridement and R = r denotes thorough debridement. To simulate the allogeneic fibular implant, the dimensions (length = 80 mm and radius = 6 mm) were obtained from the manufacturer. The axial direction of the fibula was defined by the entry point and lesion centroid. The entry point was located in the trochanteric lateral cortex of the femur. The distance of the cortical bone from the apex of the fibula was 5 mm. The remaining voids were occupied by impaction cancellous bone after the debridement.

## Results

### Stress transfer path

**Fig. 4:**
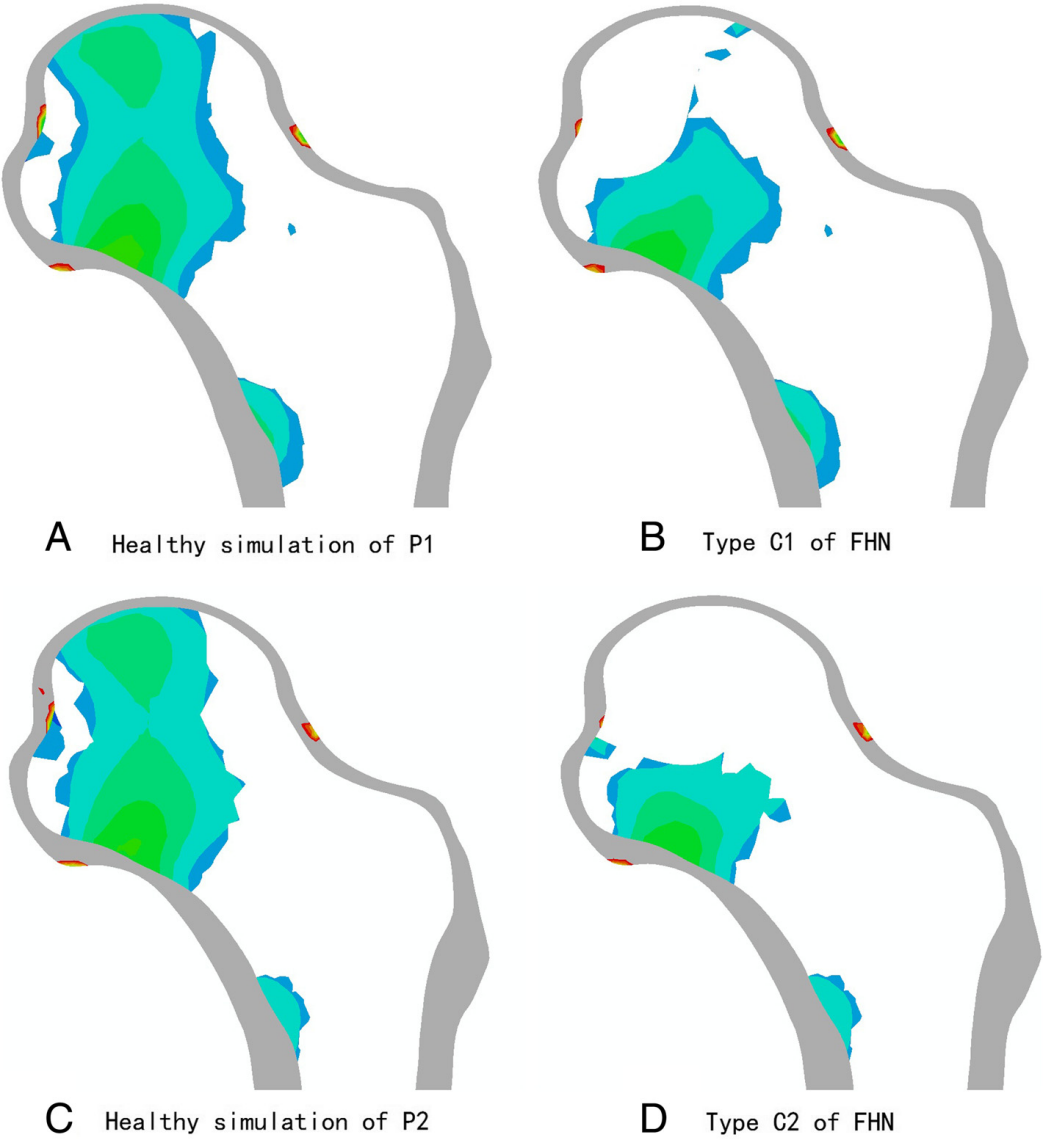
Principal stress distributions in the femoral head

The principal stress transfer characteristics are the most important biomechanical index in the performance evaluation of FHN. In all femoral heads, the principal stress transfer patterns were computed during a gait midstance. Figs. 4a and c show that the principal stress distributions in healthy conditions are from the top of the femoral head to the femoral calcar. As shown in Figs. 4b and d, the stress transfer paths are broken off, and the areas that bear the principal stress are less than approximately 50 % of the healthy simulations. The principal stress transfer efficiency markedly decreased.

### Stress of the anterolateral column

**Fig. 5:**
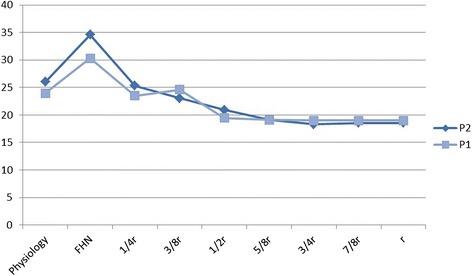
Relationship between anterolateral stress and debridement region

FAIBG presents a considerably small risk of structural collapse compared with the untreated situation. Fig. 5 shows the relationship between anterolateral stress and the debridement region. The maximum stress values were 23.95 MPa in P1 and 25.99 MPa in P2. The JIC C1 FHN stress of 30.31 MPa increased by approximately 26.56 %, which is higher than that obtained in the healthy condition (P1). The JIC C2 FHN stress of 34.58 MPa increased by approximately 33.05 %, which is higher than that obtained in the healthy condition (P2). There is an obvious stress concentration region in the anterolateral column of the necrotic femoral head. When the debridement radius was 1/4 r, the stress was 23.52 MPa in P1 and 25.31 MPa in P2, which are approximately 22.4 % and 26.81 % lower than those obtained in the JIC C1 (P1) and the JIC C2 conditions (P2), respectively. The peak stresses of the two postoperative cases returned to the near-healthy levels. After the FAIBG procedure, the stress concentration regions disappeared. When the debridement radius was greater than 3/8 r, the peak stress does not significantly change with an increase in the debridement radius.

### Peak stress of the residual necrotic bone

**Fig. 6:**
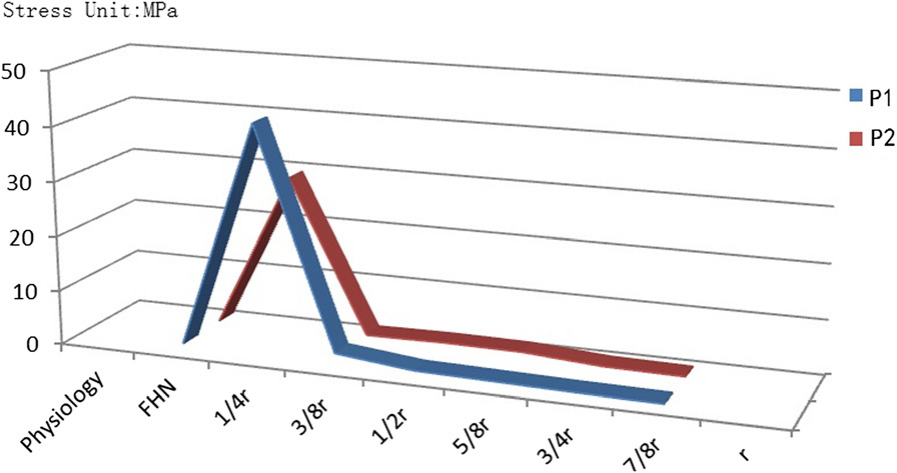
Peak stress of the residual necrotic bone

Figure 6 demonstrates that the debridement size affects the stress distribution in the residual necrotic bone. Seven different necrotic debridement sizes ranging from 1/4 r to r were selected to study the effect of the debridement radius on the residual necrotic bone. The relationship between the debridement size and the stress of the residual necrotic bone is shown in Fig. 5. When the debridement radius was 1/4 r, the peak stress increased by 3762 % and 1217 % compared with the values obtained in the JIC C1 and JIC C2 conditions, respectively. When the debridement was at least 3/8 r, the peak stress in the residual lesion rapidly decreased and returned to the physiological level.

### Efficiency of debridement

**Fig. 7:**
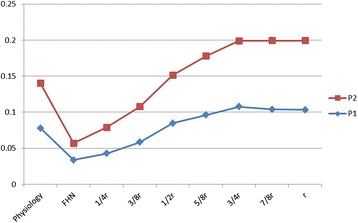
Load share ratios based on different debridement regions

The average stresses were calculated from all of the elements on the necrotic region and anterolateral column. The FAIBG procedure effectively decreased the average stress of the anterolateral column. However, a low-stress region gradually increased in the anterolateral column with an increase in the debridement radius. The load share ratio (LSR) is defined as the ratio of the average necrotic region stress to the average anterolateral column stress, which represents the bearing capacity of different material models in the femoral head. Fig. 7 displays the relationship between the necrotic region and anterolateral column based on different debridement regions. The bearing capacity of the necrotic region in the untreated condition is markedly decreased. The LSR increased with an increase in the debridement radius. In particular, when the debridement radius was larger than 1/2 r, the LSR was larger than that obtained in the normal condition, which indicates that a proper debridement region may eliminate the stress concentration, but if the debridement region is too large, it may introduce the stress-shielding phenomenon.

### Model validation

**Fig. 8:**
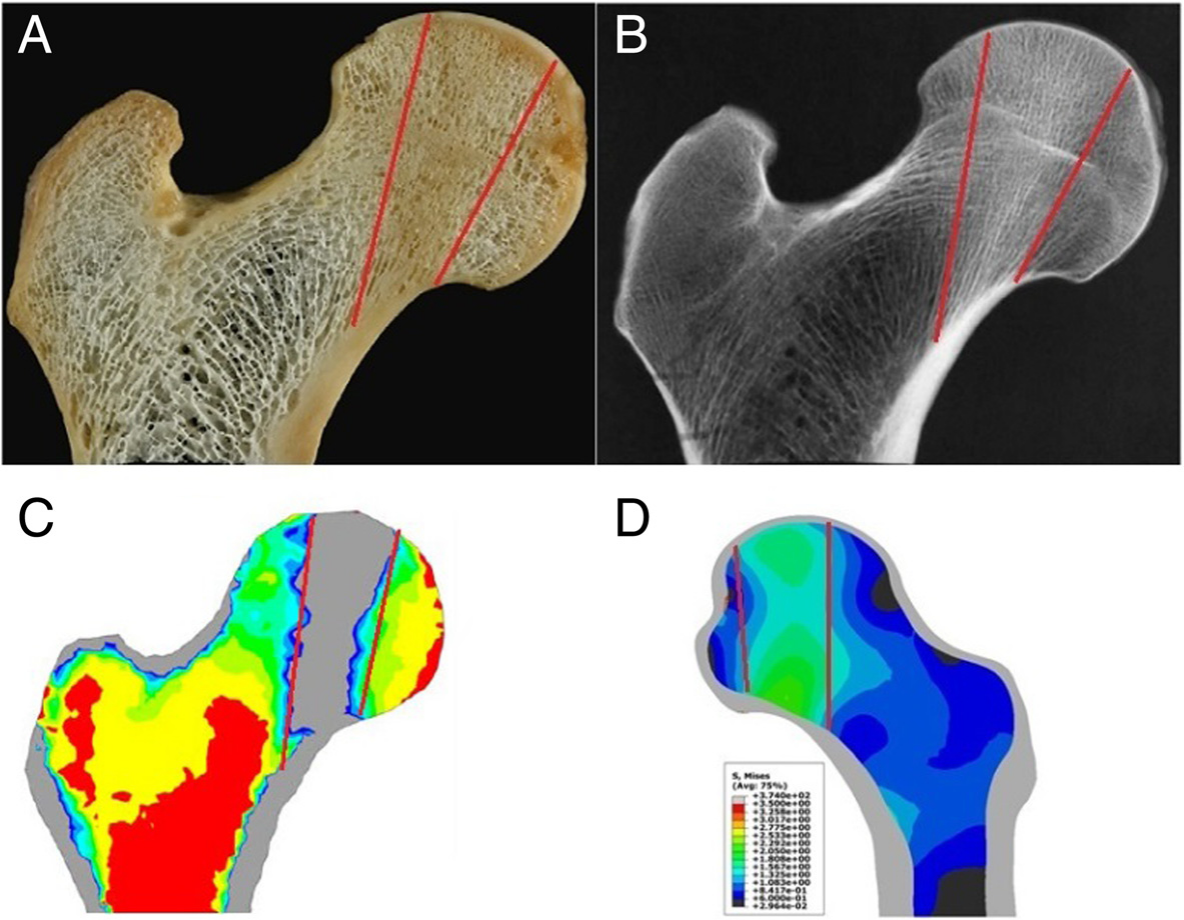
Photograph (**a**), radiograph (**b**), the previous simulation results (**c**) and the computational results (**d**) of the human proximal femur investigated in this study

The principal compressive trabecula loads the principal compressive stress of the femoral head (Figs. 8c and d), which correlates well with the bone density distribution (Fig. 8b) [17]. The shape and location of the biomechanical transfer path for both load cases are consistent with the trabecular features in the cross-sections of the cadaver bone (Fig. 8a) [18, 19]. The trabeculae in the corresponding areas are clearly thinner. Simultaneously, the simulation results of our study (Figs. 4a and c) and the results of previous studies presented in the literature [13] have strongly similar stress patterns. Hence, we hypothesize that the finite element results can reflect the physical phenomenon of the hip and evaluate the results.

## Discussion

Allograft bone is currently the most commonly used materials for the hip preserving procedure of FHN. Allograft fibula is used to provide structure and biomechanical support and impaction cancellous bone was used as biological repair material to promote osteogenesis during bone healing. The FAIBG procedure, as one of allograft bone methods, represents a proven technique to maintain the shape of the femoral head and reduce the risk of FHC in its early stages. Rosenwasser [20] first described thorough debridement and bone grafting for the treatment of FHN in 1994. This technique is an effective method for young patients with early stage FHN, which delays the progression of osteoarthrosis and subsequent HR. Tao [21] reported an 80 % clinical success rate with a mean follow-up time of 24 months among fifteen patients who had surgical therapy with thorough debridement with bone grafting. However, these procedures may cause serious artificial damage and complications because of capsulotomies or the destruction of the cortical bone of the femur neck fundus and require relatively high-cost and complicated technique. In 2008, Shi [22] reported 67 hips subjected to internal bracket implanting with partial debridement for FHN. These researchers showed a 64.2 % (43/67) success rate with an average follow-up of 23 months. In 2013, Shi [23] treated 25 patients using an allograft fibula with partial debridement for FHN and reported satisfactory results in 18 of 25 (72 %) patients with a 24-month follow-up. These minimally invasive procedures could reduce the artificial damage and complications but result in a poorer clinical outcome because they cannot provide both repaired materials and biomechanical structural support during healing of the necrosis region. FAIBG with proper debridement is an effective head-preservation method, and we achieved an average clinical success rate of 90.3 % with a mean follow-up time of 37.5 months [24]. All views are based on clinical observation experience and lack a biomechanical basis. Hence, both “thorough debridement” and “partial debridement” are not universally accepted because no compelling evidence indicates which method is better at reducing the collapse risk of the femoral head, which encourages us to apply our experiences to a computational biomechanical analysis of the extent of debridement to reduce the collapse risk of FHN.

In our study, we adopted a subject-specific computational approach to consider the changes in the stress distribution of the anterolateral cortical bone and residual necrotic bone. Fig. 4 shows that the stress transfer paths in both JIC C1 and C2 are completely broken off, which indicates that surgical intervention should be performed. The effect of the debridement size with FAIBG on the collapse risk is clearly demonstrated in Fig. 5. After FAIBG, the peak stress of the anterolateral cortical bone in all conditions could return to the physiological level, and in two cases, the decrement/increment in stress was almost less than 0.1 % when the debridement radius increased. Hence, the collapse risk of the femoral head can be effectively reduced using an allo-fibula support to bear the load. Ueo [28] reported that the concentrated stress around the residual necrotic bone may induce development of the disease. When the debridement size is at least 3/8 r, the peak stress of the residual necrotic bone also returns to the pathological level, which denotes that the progression of necrosis will not deteriorate after surgical intervention. Fig. 7 shows that a proper debridement region may eliminate the stress concentration, but if the debridement region is too large and the bone grafting provides an oversized support intensity, the stress shielding phenomenon may be introduced. According to Wolff’s law, the structure and function of bone are interdependent. Stress shielding may cause disused bone loss of the anterolateral cortical bone, which results in fracture and collapse. Our results provide specific biomechanical evidence to support the viewpoint that FAIBG with a debridement region of 3/8 - 1/2 appears to be a better choice for resisting the collapse of JIC C FHN.

Thorough debridement has been reported in previous studies [20, 21, 25–27]. However, this procedure is difficult and time-consuming and is associated with serious artificial damage. Simultaneously, thorough debridement and bone grafting may cause stress shielding, which results in fracture and collapse. FAIBG with partial debridement can eliminate stress concentration and stress shielding and ensure that the stress of the residual bone does not increase. This technique has a distinct biomechanical basis, saves time, requires relatively lower-cost and introduces a low risk of artificial damage. Hence, FAIBG without thorough debridement appears to be better than FAIBG with thorough debridement.

## Conclusions

In this study, we propose using computational biomechanical technology to explore different mechanical performances of FAIBG with and without thorough debridement in order to provide a biomechanical basis for selecting the proper treatment in clinic settings. Eighteen computational models were constructed to simulate two subtypes of FHN with seven debridement radii during the FAIBG procedure. The simulation results provide specific biomechanical evidence to support the finding that FAIBG with a debridement region of 3/8 - 1/2 appears to be a better choice for resisting the collapse of JIC C FHN. Furthermore, FAIBG without thorough debridement, which requires relatively simple technique and reduces artificial damage, appears to be a better method for resisting the collapse of JIC C1 and JIC C2 FHN. This manuscript also presents a preliminary approach to investigate the FAIBG procedure with thorough debridement, and a more detailed analysis will be reported in the near future.
